# The relationship between basic psychological needs satisfaction and personal growth initiative among female college students in Western China: a chain mediation effect

**DOI:** 10.3389/fpsyg.2025.1689035

**Published:** 2025-11-26

**Authors:** Shangxia Song, Bihua Yan

**Affiliations:** School of Teacher Development, Shaanxi Normal University, Xi’an, China

**Keywords:** female college students in Western China, basic psychological needs satisfaction, psychological capita, future time perspective, personal growth initiative

## Abstract

**Introduction:**

Focusing on the growth motivation of female college students in the resource-constrained environments of Western China, this study integrates perspectives from Self-Determination Theory and Positive Psychology to examine the relationship between basic psychological needs satisfaction and personal growth initiative, as well as the underlying mechanisms.

**Methods:**

Based on convenience sampling, a survey was conducted among 473 female college students with household registration in Western China who were studying at universities in the same region (*M* = 20.18, SD = 1.26; 70.6% were from rural areas). The survey employed the Basic Psychological Needs Scale, Personal Growth Initiative Scale, Positive Psychological Capital Questionnaire, and Future Time Perspective Scale.

**Results:**

This study found that among female college students in Western China, basic psychological needs satisfaction significantly and positively predicted psychological capital, future time perspective, and personal growth initiative. Psychological capital and future time perspective significantly and positively predicted personal growth initiative. Mediation analysis revealed that psychological capital and future time perspective played a chain mediation role between basic psychological needs satisfaction and personal growth initiative among female college students in Western China.

**Discussion:**

This study reveals key mechanisms of personal growth among female college students in Western China within resource-limited environments. Basic psychological needs satisfaction can enhance personal growth initiative through multiple mediation pathways involving psychological capital and future time perspective. This provides a theoretical basis and practical direction for targeted interventions and subsequent empirical research. Education policies should focus on fulfilling students’ needs for autonomy, competence, and relatedness, while mental health services should attend to students’ levels of psychological capital and future time perspective.

## 1 Introduction

The problem of unbalanced and inadequate development in Western China remains prominent (Liu et al., 2024). Female college students in Western China face multiple challenges in their growth and development. First, the underdeveloped regional economy has resulted in significantly inferior access to educational resources compared to eastern regions (Li and Xue, 2022), thereby directly constrains their academic performance. The predicament of resource scarcity forces them to cope with equal or even greater academic demands and career competition pressures under disadvantaged conditions, which persistently undermines their academic self-confidence and career competitiveness. Second, the distortion of gender role expectations within traditional cultural perspectives imposes multifaceted constraints on female college students’ personal development. Benevolent sexism, manifested as “needing protection” may implicitly cast doubt on their individual capabilities (Yang et al., 2021). Meanwhile, the invisible pressure of “family responsibility first” restricts their pursuit of higher personal achievement (Chen et al., 2022). Third, when building social relationships, women must endeavor to integrate into male-dominated academic or social groups (Kim and Kim, 2021) while simultaneously confronting competitive pressures from female peers (Wang et al., 2021). This coexistence of assimilation demands and competition pressures impedes the establishment of authentic, supportive, and reciprocal relationships. Collectively, these three dilemmas erode the foundation for satisfying the basic psychological needs of female college students in Western China, thereby severely constraining their motivation for self-development.

According to Self-Determination Theory, basic psychological needs consist of the needs for relatedness, competence, and autonomy (Ryan and Deci, 2000a). The need for relatedness manifests through an individual’s experience of acceptance, support, and emotional connectedness in interpersonal interactions. The need for competence arises from the perception of growth in one’s efficacy and capabilities through interactions with the environment. The need for autonomy is expressed as congruence between behavior and internal values, rather than passive compliance driven by external pressures (Ryan and Deci, 2000a). The theory further elucidates that basic psychological needs satisfaction is key to stimulating an individual’s intrinsic growth orientation: when these needs are met, they yield broad positive outcomes such as heightened intrinsic motivation, positive emotions, mental health, and increased well-being (Deci and Ryan, 2000). while also prompting individuals to engage more actively in the process of self-growth (Deci and Ryan, 2012; Ryan and Deci, 2000b). This proactive state of growth conceptually aligns closely with the typical manifestations of personal growth initiative (Robitschek, 1998; Weigold et al., 2013). Personal growth initiative is a pivotal construct in positive psychology. It refers to an individual’s proactive and deliberate tendency to improve themselves during developmental processes, including both cognitive and behavioral aspects (Robitschek, 1998). Subsequent research has proposed a four-dimensional framework of personal growth initiative: which includes Readiness for Change, Using Resources, Planfulness, and Intentional Behavior. These dimensions are interrelated and jointly promote personal growth (Robitschek et al., 2012; Robitschek and Keyes, 2009). The goal-setting, resource-seeking, and self-directed actions embodied by these dimensions are precisely the external behavioral manifestations that emerge once basic psychological needs—particularly the need for autonomy and the need for competence—are satisfied. Empirical evidence substantiates that individuals with a high level of personal growth initiative demonstrate superior adaptability and positive outcomes across multiple domains, including academic achievement, career advancement, mental health, and interpersonal relationships (Cai and Lian, 2022; Weigold et al., 2024). For example, personal growth initiative plays a mediating role between entrepreneurial learning and entrepreneurial intentions (Singh et al., 2025), and can also moderate the mediating pathways from fear of COVID-19 to life satisfaction, as well as from preventive behaviors to life satisfaction (Green and Yıldırım, 2022). This finding empirically validates the Self-Determination Theory proposition that basic psychological needs satisfaction serves as a fundamental cornerstone for promoting healthy individual development (Barański and Poprawa, 2024; Deci and Ryan, 2000). Consequently, targeted enhancement of personal growth initiative levels can promote well-rounded human development (Gong et al., 2022; Jiao et al., 2024).

Basic psychological needs satisfaction is closely related to personal growth initiative (Weigold et al., 2021). According to Self-Determination Theory, each basic psychological need independently predicts various indicators of psychological growth, internalization processes, and well-being (Slemp et al., 2024; Van den Broeck et al., 2016). Specifically, satisfaction of autonomy and relatedness needs directly predicts autonomous motivation, while satisfaction of the need for competence directly predicted meaningful work (Autin et al., 2021). This indicates that basic psychological needs satisfaction provides a robust foundation for personal growth, thereby increasing individuals’ propensity to proactively pursue development. Research on college students indicates that satisfaction of autonomy and competence needs positively correlates with personal growth initiative (Negovan and Bogdan, 2013). Simultaneously, satisfaction of relatedness and competence needs is directly and positively associated with well-being. When students perceive their courses support autonomy, they exhibit higher autonomous motivation (Zhu et al., 2024). Autonomous motivation is associated with positive emotions and good performance, and can improve mental health and efficacy (Deci and Ryan, 2008). High levels of autonomous motivation constitute a critical driver of personal growth. For college students, basic psychological needs satisfaction constitutes a critical pathway to promoting academic engagement and personal growth (Li et al., 2025; Schönfeld and Mesurado, 2025). Relevant research using Partial Least Squares Structural Equation Modeling (PLS-SEM) has found that basic psychological needs satisfaction can mediate the relationship between multiple psychological variables and college students’ willingness to use online learning platforms (Deng et al., 2024). When college students experience perceived mastery over academic tasks (competence needs satisfaction), exercise choice and decisional autonomy in learning (autonomy needs satisfaction), and establish positive relationships with teachers and peers (relatedness needs satisfaction), they demonstrate deep engagement in learning. This encompasses proactively setting goals, actively exploring knowledge, and persistently overcoming challenges. Such intrinsic motivation directly promotes the development of personal growth initiative (Holzer et al., 2021; Li et al., 2025).

### 1.1 Basic psychological needs satisfaction and personal growth initiative

Based on Self-Determination Theory, existing research has thoroughly elucidated the pivotal role of basic psychological needs satisfaction in fostering personal growth initiative (Van den Broeck et al., 2016), which provides important theoretical and empirical foundations for understanding the personal development of college students. However, research on personal growth initiative among female college students is markedly insufficient, resulting in limited understanding of this group’s unique developmental pathways and psychological processes. The existing quantitative studies have included gender in their analyses, but it is predominantly treated as a control variable rather than a core variable for in-depth examination, this approach makes it difficult to reveal the specific psychological motivations, internal experiences, and behavioral tendencies of female college students regarding the satisfaction of their basic psychological needs for autonomy, competence, and relatedness during their personal growth process. As a result, the theoretical explanatory power regarding the developmental complexity of specific groups is limited. For example, a study verifying the Personal Growth Initiative Scale-II among Iranian adolescents reported gender invariance characteristics of the scale. However, as its primary objective was scale validation, the research did not extend to examining the causes and underlying mechanisms of gender differences (Habibi Asgarabad et al., 2025). Another study included gender comparisons when exploring the relationships among academic self-efficacy, personal growth initiative, and learning engagement, and the results demonstrated that there was no significant difference in the mediating effect across genders (Gülşen and Şahin, 2023). More importantly, research focusing on female college students in Western China remains scarce, which is in stark contrast to the multiple structural challenges they confront. The disadvantaged context faced by female college students in Western China tends to inhibit the satisfaction of their basic psychological needs for autonomy, competence, and relatedness, and the fulfillment of these needs plays a pivotal role in fostering personal growth initiative. Therefore, in-depth investigation into the influencing factors, mechanisms, and pathways for improving personal growth initiative is particularly important for understanding their unique developmental dynamics within disadvantaged groups.

### 1.2 The mediating role of psychological capital

Psychological capital represents a positive psychological state during individual growth and developmental processes, comprising four positive psychological resources: self-efficacy, hope, optimism, and resilience (Luthans and Youssef, 2004). These components enable individuals to effectively overcome challenges during growth, enhance emotional regulation and stress resistance (Luthans et al., 2004). A study conducted among Turkish youth found that love of life significantly promotes flourishing through the mediating effects of optimism and hope (Yıldırım et al., 2024b). Meanwhile, research on Syrian refugees demonstrated that resilience and hope directly predict life satisfaction and flourishing, while also playing significant roles beyond social support and belongingness (Yıldırım et al., 2022). Psychological capital safeguards against depressive symptoms and enhances mental health among college students (Luo et al., 2023). It also mediates the relationship between social capital and career adaptability in this population (Gheyassi and Alambeigi, 2024). When confronting learning or life challenges, individuals with higher levels of psychological capital are more likely to adopt proactive problem-solving strategies (Chen et al., 2024). Psychological capital is easily influenced by external environment, indicating its plasticity, and can be effectively improved through training and interventions (Luthans and Youssef-Morgan, 2017). Recently, a study on Chinese science and engineering teachers revealed the partial mediating role of psychological capital between self-directed learning and interdisciplinary teaching ability using PLS-SEM (Su et al., 2025). Relevant empirical studies have found that psychological capital plays a mediating role between intolerance of uncertainty and job satisfaction as well as work performance (Yıldırım et al., 2024a). The formation of psychological capital is closely linked to basic psychological needs satisfaction, which serves as a key factor in the accumulation of psychological capital (Carmona-Halty et al., 2019). Basic psychological needs satisfaction serves as the fundamental environmental condition for activating individuals’ intrinsic motivation (Deci and Ryan, 2000), which can be transformed into stable positive psychological resources, thereby promoting proactive growth behaviors. When college students’ needs for competence, autonomy, and relatedness are satisfied, they are more likely to cultivate positive psychological resources such as hope, optimism, resilience, and self-efficacy (Schönfeld and Mesurado, 2025). This process consequently stimulates learning motivation, enhances academic engagement, and improves educational performance (Salikhova et al., 2024). Simultaneously, psychological capital has been demonstrated to facilitate personal growth initiative (Meyers et al., 2015). As a powerful internal driving force for confronting challenges and promoting development, it motivates individuals to proactively seek growth opportunities, effectively overcome obstacles, and strengthen confidence in their ability to grow. This, in turn, enhances their capacity for active planning and achievement of personal development, ultimately promoting sustained growth and advancement.

### 1.3 The mediating role of future time perspective

Future time perspective represents a malleable cognitive-motivational construct that focuses on an individual’s capacity and propensity to anticipate and plan for the future (Kooij et al., 2018). Future time perspective reflects an individual’s universal concerns about the future and corresponding cognitive processes, while also highlighting how individuals actively adjust their motivations and behaviors across different life stages based on their perception of future time (Kooij et al., 2013). There is divergence in the academic community regarding the evaluation methods and dimensional divisions of future time perspective. However, through a meta-analysis, Kooij et al. (2018) noted that its core characteristics can be distilled into three key dimensions: future orientation, continuity, and affectivity. Song (2004) classified future time perspective into five dimensions: behavioral commitment, future efficacy, long-term goal orientation, purpose consciousness, and future imagery. From the perspective of motivational mechanisms, the formation and stimulation of future time perspective can be deeply explained by Self-Determination Theory. Specifically, when students’ need for autonomy is satisfied, their perceived control over personal behaviors and choices increases, thereby enhancing their propensity to set and pursue long-term learning goals (Zhu et al., 2024). Competence needs satisfaction equips students with confidence in facing future challenges (Shin and Gwon, 2023), thereby fostering greater willingness to engage in long-term goals that demand sustained effort investments. Changes in students’ relatedness need satisfaction is positively correlated with changes in learning goal orientation (Janke, 2022).

When both basic psychological needs satisfaction and future time perspective are high, individuals experience stronger positive effects on health behaviors (Visser and Hirsch, 2014). Individuals with a high level of future time perspective demonstrate stronger goal orientation and planning capabilities. They can clearly perceive future needs, establish systematic objectives, and formulate implementation strategies. These individuals tend to link current behavior with future goals, perceiving present efforts as critical pathways to future success (Husman and Shell, 2008; Li, 2021). When individuals clearly perceive the significance of their current actions for future goals, their motivation is significantly enhanced (Simons et al., 2004). In academic contexts, students with high future time perspective not only exhibit sustained motivation toward long-term objectives, but also activate the execution of short-term goals by anticipating the future utility of classroom activities. Consequently, they demonstrate stronger academic persistence in their studies (Husman and Lens, 1999). Therefore, this integrative cognitive framework based on future goals essentially constitutes a critical pathway through which basic psychological needs promote individuals’ development via future time perspective.

### 1.4 The chain mediating effects of psychological capital and future time perspective

Previous research indicates that individuals with higher psychological capital demonstrate greater confidence and are more proactive in perceiving, planning, and shaping their future, thereby significantly enhancing their future time perspective (Elrehail et al., 2021; Schelleman-Offermans and Massar, 2020). Psychological capital, as a positive psychological resource, can effectively enhance individuals’ proactiveness in the face of adversity and encourage them to actively anticipate the future (Abubakar et al., 2019; Song et al., 2021). Specifically, individuals with high self-efficacy hold positive assessments of their own capabilities and view future problems as solvable challenges. Optimism guides individuals to attribute future events positively, interpreting setbacks as temporary and surmountable while maintaining confidence that their efforts will bring positive results. Hope prompts individuals to establish longer-term goals and actively identify effective pathways to attain them. Resilience enables individuals to recover quickly, adapt effectively, and persistently progress when confronting future adversities and challenges (Luthans and Youssef-Morgan, 2017). Psychological capital can significantly and positively predict positive future expectations. Positive future expectations refer to an individual’s degree of optimism and hope regarding their personal future (Yıldırım et al., 2023), encompassing a positive outlook on what is to come. This aligns with the emphasis future time perspective places on an individual’s focus toward the future. Thus, the four core dimensions of psychological capital collectively enhance individuals’ future time perspective, motivating them to perceive, connect, and shape the future in a constructive and forward-looking manner. As previously indicated, individuals with higher levels of psychological capital demonstrate a stronger propensity to proactively seek opportunities and pathways for personal growth, exhibiting heightened personal growth initiative (Meyers et al., 2015). Concurrently, individuals with higher levels of future time perspective tend to set long-term goals, formulate implementation plans and sustain investment, which closely aligns with the core construct of personal growth initiative (Lens et al., 2012). Therefore, the satisfaction of basic psychological needs can not only directly promote the development of personal growth initiative but also promote individual proactive growth by nurturing psychological capital and enhancing future time perspective.

### 1.5 The present study

**Figure 1:**
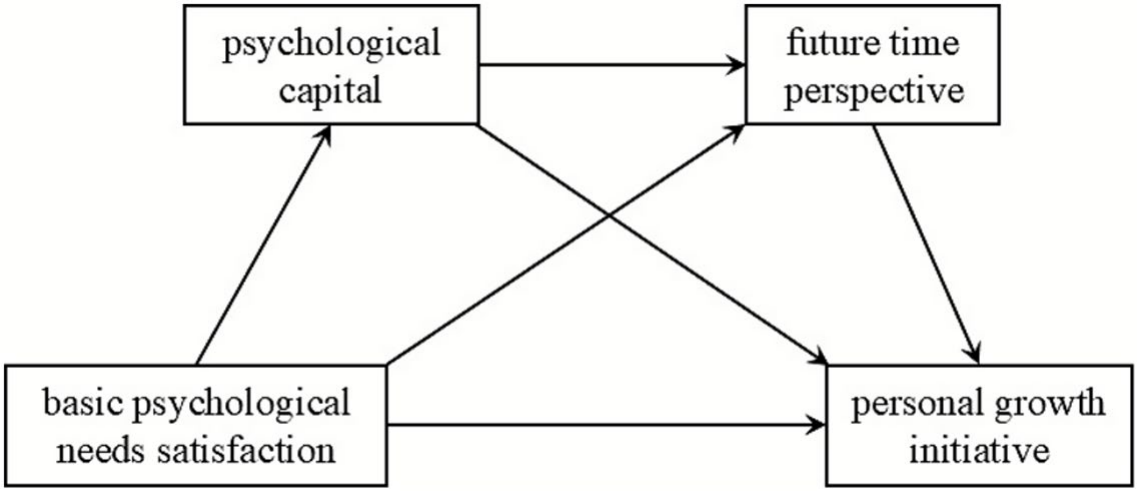
Hypothesized model.

Based on self-determination theory and the perspective of positive psychology, this study constructed a chain mediation model to examine the underlying mechanism between basic psychological needs satisfaction and personal growth initiative among female college students in western China. Existing research indicates that, basic psychological needs satisfaction is a key factor in the accumulation of psychological capital (Carmona-Halty et al., 2019). When students’ basic psychological needs are satisfied, they are more likely to develop positive psychological capital (Schönfeld and Mesurado, 2025). Individuals with higher levels of psychological capital tend to perceive, plan, and shape their future with greater confidence and proactivity, thereby significantly enhancing their future time perspective (Elrehail et al., 2021; Schelleman-Offermans and Massar, 2020). At the same time, they are more inclined to actively seek out and engage in personal growth activities (Meyers et al., 2015). Some studies have also explored the effect of basic psychological needs satisfaction on personal growth initiative (Negovan and Bogdan, 2013). Recently, research has shown that future time perspective significantly predicts personal growth initiative (Zhu et al., 2025). Building on the above research, this study integrates these mechanisms into a comprehensive chain mediation model. (the model is shown in Figure 1). We aim to reveal the complete internal process through which basic psychological needs satisfaction cultivates individuals’ psychological capital, which in turn enhances their future time perspective, and ultimately promotes their personal growth initiative.

Based on this, the study proposes the following four hypotheses:

*H1*: Among female college students in Western China, basic psychological needs satisfaction significantly and positively predict personal growth initiative.

*H2*: Among female college students in Western China, psychological capital mediates the relationship between basic psychological needs satisfaction and personal growth initiative.

*H3*: Among female college students in Western China, future time perspective mediates the relationship between basic psychological needs satisfaction and personal growth initiative.

*H4*: Psychological capital and future time perspective exert a chain mediation effect between basic psychological needs satisfaction and personal growth initiative.

## 2 Methods

### 2.1 Participants and procedure

#### 2.1.1 Participants

This study targeted female college students with registered residence in Western China who were studying at universities in Western China were selected as study participants. Of the 520 questionnaires distributed, 473 were returned as valid, yielding a valid response rate of 90.96%. Among the participants, 135 were first-year undergraduates (28.5%), 194 were second-year undergraduates (41.0%), and 144 were third-year undergraduates (30.4%). Additionally, 334 participants (70.6%) were from rural areas, and 139 (29.4%) were from urban areas. The average age of the participants was 20.18 years (SD = 1.26). Fourth-year undergraduates, who are in the graduation period, were not included in the sample due to significantly decreased willingness to participate and limited time investment. This decision was made for feasibility considerations.

#### 2.1.2 Procedure

A convenience sampling method was employed, and data were collected through an online platform. After explaining the research purpose, survey content, and principles of confidentiality, informed consent was obtained from all respondents. Confidentiality and anonymity of participant data were ensured. This study received ethical approval.

### 2.2 Measures

#### 2.2.1 Basic psychological needs scale

The Basic Psychological Needs Scale, originally developed by Gagné (2003) and revised by Li (2009), was employed. The Chinese version comprises 21 items and consists of three dimensions: Autonomy, Competence, and Relatedness. A sample item is: “I feel I can autonomously decide how to live my life; my life is governed by my own choices.” Responses were recorded on a 7-point Likert scale ranging from 1 (completely disagree) to 7 (completely agree). A higher total score indicates a greater degree of satisfaction of basic psychological needs. In the current study, the Cronbach’s alpha coefficient for the scale was 0.871, the model fit indices were $\chi^{2}/\mathrm{df}=1.34$ , RMSEA = 0.03, CFI = 0.98, TLI = 0.98.

#### 2.2.2 Positive psychological capital questionnaire

The Chinese version of the Positive Psychological Capital Questionnaire, developed by Zhang et al. (2010), was employed. This scale comprises 26 items measuring four dimensions: Self-Efficacy, Resilience, Hope, and Optimism. A sample item is: “Many people appreciate my talents.” Responses were recorded on a 7-point Likert scale (1 = “completely disagree” to 7 = “completely agree”). The higher the total score, the higher the individual’s level of positive psychological capital. The Cronbach’s alpha coefficient for this questionnaire in the present study was 0.935, the model fit indices were $\chi^{2}/\mathrm{df}=1.56$ , RMSEA = 0.03, CFI = 0.98, TLI = 0.98.

#### 2.2.3 Future time perspective scale

The Chinese version of the General Future Time Perspective Questionnaire, developed by Song (2004), was employed. This scale consists of 20 items (with items 2, 17, 18, 19, and 20 being reverse-scored), assessing five dimensions: Behavioral Commitment, Future Efficacy, Long-Term Goal Orientation, Purpose Consciousness, and Future Imagery. A sample item is: “I have daily goals that I work toward.” Responses were recorded on a 4-point Likert scale, ranging from 1 (completely agree) to 4 (completely disagree). During the data processing phase, reverse-scoring was first applied to the negatively worded items, followed by reverse-scoring of all items to ensure higher scores indicate more positive tendencies. Ultimately, a higher final score represents a stronger level of an individual’s future time perspective. The Cronbach’s alpha coefficient for this scale in the present study was 0.933, the model fit indices were $\chi^{2}/\mathrm{df}=2.42$ , RMSEA = 0.05, CFI = 0.97, TLI = 0.96.

#### 2.2.4 Personal growth initiative scale

The Personal Growth Initiative Scale-II originally developed by Robitschek et al. (2012) and adapted by Tian (2016), was employed. The Chinese revised version comprises 16 items and consists of four dimensions: Readiness for Change, Planfulness, Using Resources, and Intentional Behavior. A sample item includes: “I have set realistic goals for how to change myself.” Responses were recorded on a 6-point Likert scale ranging from 0 = “strongly disagree” to 5 = “strongly agree.” A higher total score indicates a higher level of personal growth initiative. The Cronbach’s alpha coefficient for this scale in the present study was 0.929, the model fit indices were $\chi^{2}/\mathrm{df}=2.58$ , RMSEA = 0.06, CFI = 0.98, TLI = 0.97.

### 2.3 Statistical methods

This study utilized SPSS 22.0 and AMOS 22.0 software for data analysis. Initially, SPSS was employed to conduct tests for common method bias, descriptive statistics, and correlation analysis. Subsequently, AMOS 22.0 was used to establish structural equation modeling and fit the chain mediation effect model. The mediation effects were tested using the Bootstrap method with 5,000 resamples to calculate 95% confidence intervals. The Bootstrap method was selected as it does not rely on assumptions of normal sampling distribution and demonstrates higher statistical power for testing mediation effects. A significant effect is indicated if the confidence interval does not include zero.

## 3 Results

### 3.1 Common method bias test

In this study, measures such as anonymity and reverse-scoring of certain items were employed as procedural controls for common method bias. Common method bias was assessed using Harman’s single-factor test method. The analysis yielded 13 factors with eigenvalues greater than 1, with the first factor explaining 34.42% of the variance, which is below the critical threshold of 40%. This indicates that no serious common method bias was present in this study.

### 3.2 Correlation analysis among variables

Descriptive statistics and correlation analyses were conducted for all variables, with the results presented in Table 1. The correlation analysis revealed significant positive correlations among basic psychological needs satisfaction (*M* = 98.82, SD = 15.70), psychological capital (*M* = 123.53, SD = 20.45), future time perspective (*M* = 52.04, SD = 10.59), and personal growth initiative (*M* = 55.50, SD = 12.34) (*r* = 0.432 ~ 0.745, *p* < 0.01). Notably, the strongest correlation was observed between basic psychological needs satisfaction and psychological capital (*r* = 0.745). This study calculated the variance inflation factor (VIF) and tolerance for all variables in the model. As shown in Table 2. The VIF values for all variables were below the empirical threshold of 5 (or the stricter threshold of 3), and the tolerances were all greater than 0.1. These results demonstrate that despite the relatively high correlation between basic psychological needs satisfaction and psychological capital, no severe multicollinearity issues were present in the model.

**Table 1:** Means, standard deviations, and correlations among variables (*n* = 473).

Variables	*M* ± *SD*	1	2	3	4
1. BPNS	98.82 ± 15.70	—			
2. PsyCap	123.53 ± 20.45	0.745^**^	—		
3. FTP	52.04 ± 10.59	0.432^**^	0.490^**^	—	
4. PGI	55.50 ± 12.34	0.607^**^	0.723^**^	0.461^**^	—

BPNS, Basic Psychological Needs Satisfaction; PsyCap, Psychological Capital; FTP, Future Time Perspective; PGI, Personal Growth Initiative. ^*^p < 0.05, ^**^p < 0.01. Same below.

**Table 2:** Tolerance and VIF for all variables.

Variables	Tolerance	VIF
BPNS	0.439	2.279
PsyCap	0.410	2.441
FTP	0.750	1.334

### 3.3 Mediation effect test

Structural equation modeling was conducted using AMOS 22.0 to examine the mediating effects of psychological capital and future time perspective in the relationship between basic psychological needs satisfaction and personal growth initiative. The fit indices for the theoretical model were: *χ*^2^ = 587, *df* = 129, *χ*^2^/*df* = 4.55, RMSEA = 0.086, CFI = 0.938, TLI = 0.926, NFI = 0.922. Both construct validity and convergent validity were reported, as shown in Table 3. The various indicators in the study demonstrate that the model exhibits acceptable fit.

**Table 3:** Construct validity and convergent validity.

Construct	Item	Factor loading	CR	AVE	Cronbanch’s *α*
BPNS	AUT	0.848	0.877	0.704	0.871
	COMP	0.839			
	REL	0.830			
PsyCap	SE	0.849	0.88	0.656	0.935
	RES	0.530			
	HOPE	0.899			
	OPT	0.902			
FTP	BC	0.905	0.936	0.748	0.933
	FE	0.906			
	LTGO	0.932			
	PC	0.683			
	FI	0.875			
PGI	RC	0.954	0.954	0.839	0.929
	PL	0.937			
	UR	0.934			
	IB	0.834			

AUT, Autonomy; COMP, Competence; REL, Relatedness; SE, Self-Efficacy; RES, Resilience; HOPE, Hope; OPT, Optimism; BC, Behavioral Commitment; FE, Future Efficacy; LTGO, Long-Term Goal Orientation; PC, Purpose Consciousness; FI, Future Imagery; RC, Readiness for Change; PL, Planfulness; UR, Using Resources; IB, Intentional Behavior. Same below.

**Figure 2:**
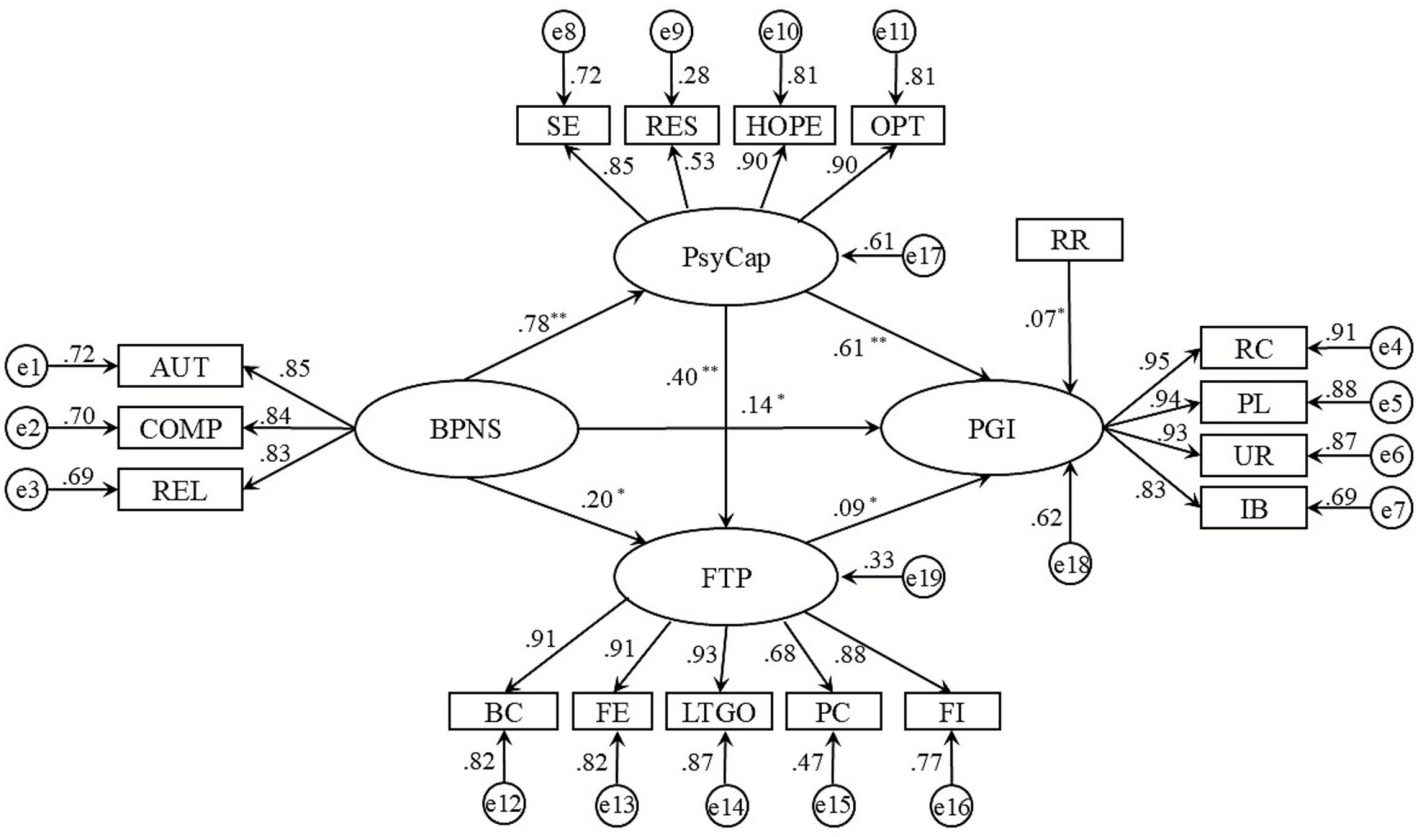
Standardized structural equation model.

Figure 2 presents a standardized structural equation model based on standardized parameter estimates, illustrating the relationships between basic psychological needs satisfaction, psychological capital, future time perspective, and personal growth initiative. Simultaneously, registered residence (RR) positively predicts personal growth initiative. Each path in the figure represents a specific pathway of influence, with the corresponding standardized coefficients indicating the strength and direction of each path relationship.

Table 4 presents the unstandardized path coefficients, standard errors, and significance levels among the variables in the structural equation model. The results indicate that basic psychological need satisfaction significantly predicts psychological capital, and both basic psychological need satisfaction and psychological capital significantly predict future time perspective. Furthermore, basic psychological need satisfaction, psychological capital, and future time perspective collectively significantly predict personal growth initiative. These findings support hypothesis H1.

**Table 4:** Unstandardized path coefficients between variables.

Pathway	Estimate	S.E.	C.R.	*p*
BPNS → PsyCap	0.871	0.054	16.016	< 0.01
PsyCap → FTP	0.270	0.052	5.144	< 0.01
BPNS → FTP	0.150	0.059	2.539	< 0.05
BPNS → PGI	0.149	0.067	2.224	< 0.05
PsyCap → PGI	0.587	0.063	9.3332	< 0.01
FTP → PGI	0.135	0.574	2.343	< 0.05

The mediation path analysis of basic psychological need satisfaction on personal growth initiative is shown in Table 5. Using the bias-corrected bootstrap method (Hayes, 2009), 5,000 resampling repetitions were performed to generate 95% confidence intervals. The results indicate that the 95% confidence intervals for both the direct and mediating paths from basic psychological need satisfaction to personal growth initiative do not include zero, demonstrating that the effects are statistically significant. The mediation effect accounted for 79.1% of the total effect of basic psychological needs satisfaction on personal growth initiative. Specific path analyses are as follows:

Path 1: BPNS → PsyCap → PGI, with an effect size of 0.511, 95% CI [0.394, 0.655]. This path is statistically significant, indicating the mediating role of psychological capital in the relationship between basic psychological needs satisfaction and personal growth initiative.
Path 2: BPNS → FTP → PGI, with an effect size of 0.020, 95% CI [0.003, 0.053]. This statistically significant path demonstrates the mediating role of future time perspective in the relationship between basic psychological needs satisfaction and personals growth initiative.
Path 3: BPNS → PsyCap → FTP → PGI, with an effect size of 0.032, 95% CI [0.007, 0.065]. This statistically significant chain mediation path confirms the chained mediating effects of psychological capital and future time perspective in the relationship between basic psychological needs satisfaction and personal growth initiative.

**Table 5:** Mediation path analysis of the effect of basic psychological need satisfaction on personal growth initiative.

Pathway	Estimate	95% CI
BPNS → PGI	0.149	[0.004, 0.293]
BPNS → PsyCap → PGI	0.511	[0.394, 0.655]
BPNS → FTP → PGI	0.020	[0.003, 0.053]
BPNS → PsyCap → FTP → PGI	0.032	[0.007, 0.065]

Above results support research hypotheses H2, H3, and H4.

## 4 Discussion

### 4.1 The direct effect of basic psychological needs satisfaction on personal growth initiative

The results indicate a positive correlation between basic psychological needs satisfaction and personal growth initiative among female college students in Western China. After controlling for the effects of mediating variables, basic psychological needs satisfaction continued to exert a significant direct positive effect on personal growth initiative, constituting 20.9% of the total effect. This finding aligns with the core viewpoints of Self-Determination Theory, which posits that satisfaction of individuals’ needs for autonomy, competence, and relatedness fosters stronger intrinsic growth motivation and positive developmental states (Deci et al., 2017; Marshik et al., 2017). The significance of this study lies in its revelation of the specific manifestations and profound implications of this theoretical mechanism among the specific group of female college students in Western China. Traditional cultural concepts, relatively limited educational resources, and the level of economic development in Western China may collectively suppress the satisfaction of basic psychological needs among this group. However, the findings of this study demonstrate that once these needs are supported and fulfilled, they become a pivotal driving force for promoting personal growth initiative among female college students in Western China. The significance of the direct effect indicates that, beyond the indirect influence mediated by other variables, there exists a more fundamental pathway through which basic psychological need satisfaction drives personal growth initiative. This may reflect a particularly strong intrinsic desire for autonomous decision-making, competence validation, and emotional support among this group in challenging environments. Specifically, the satisfaction of autonomy needs may mean that when they receive more respect and support in their choice of major, career planning, and even life decisions—rather than being constrained by traditional gender roles or family expectations—their willingness to actively shape their personal future is fundamentally enhanced. The satisfaction of competence needs may help them overcome self-doubt stemming from disparities in basic educational resources; by accumulating successful academic and social experiences, they gain confidence in their abilities, thereby empowering them to set and pursue long-term goals. Meanwhile, the satisfaction of relatedness needs can provide an emotional buffer in unfamiliar academic environments, giving them the courage to explore proactively and grow with initiative.

### 4.2 Examination of the independent mediating effects of psychological capital and future time perspective

This study validated the independent mediating role of psychological capital between basic psychological needs satisfaction and personal growth initiative. This pathway represents the primary mediating route, accounting for 71.8% of the total effect. This not only confirms the core mediating role of psychological capital but also reveals the key psychological process through which intrinsic psychological needs are transformed into growth-oriented actions among resource-constrained female college students in Western China. According to Self-Determination Theory, basic psychological needs satisfaction fosters the accumulation of psychological capital through three mechanisms: competence need satisfaction enhances self-efficacy (Shin and Gwon, 2023); autonomy need satisfaction strengthens psychological resilience (Zhang et al., 2025); and relatedness need satisfaction cultivates optimism and hope. Research indicates that the four dimensions of psychological capital (hope, optimism, resilience, and self-efficacy) constitute core resources that promote adolescents’ mental health and play a protective role in stressful situations (Yıldırım et al., 2025). Meanwhile, social support and resilience mediate the impact of stress on life satisfaction (Yıldırım and Green, 2023). In the context of this study, for female college students in Western China, competence need satisfaction often signifies overcoming initial disadvantages caused by educational resource disparities; autonomy need satisfaction enables them to develop a sense of agency over personal development, forming the foundation for psychological resilience; while relatedness need satisfaction — particularly through establishing support from mentors and peers in unfamiliar environments far from home — provides the indispensable emotional foundation for them to confidently maintain optimism about the future and nurture hope. Furthermore, psychological capital drives growth-oriented behaviors: self-efficacy promotes active learning engagement; resilience sustains long-term investment and facilitates proactive coping with challenges; and hope and optimism collectively inspire future-oriented planning and exploratory endeavors (Chen, 2024; You, 2016), resilience and hope play a mediating role in the impact of academic burnout on well-being (Cengiz et al., 2025). Focusing on female college students in resource-constrained western regions, the results revealed a particularly pronounced advantage in the conversion of psychological resources under conditions of basic psychological needs satisfaction. Specifically, basic psychological needs satisfaction serves as a highly significant positive predictor of psychological capital, while psychological capital strongly drives personal growth initiative. This indicates that among female college students in Western China, the accumulation of psychological capital through basic psychological needs satisfaction represents one of the most effective mechanisms for stimulating personal growth initiative under disadvantaged conditions.

This study simultaneously identified a significant mediating effect of future time perspective between basic psychological needs satisfaction and personal growth initiative. The effect size is small, accounting for only 2.8% of the total effect. However, this finding may have unique practical significance in special groups and warrants further exploration. The resource constraints in Western China predispose female college students to adopt a short-term goal orientation, such as early employment and part-time jobs. However, the pathway of “BPNS → FTP” can help them break the cognitive myopia caused by environmental factors and rebuild their ability to plan for long-term goals by improving the clarity of future goals. Resource scarcity also tends to induce survival pressure characterized by “reproducing reality over ideals” (Gu et al., 2020), which can impede the immediate enactment of future-oriented behaviors. Future time perspective functions as a distal regulatory factor within psychological functioning, its impact on personal development may unfold progressively over time. Furthermore, for individuals with lower levels of psychological capital, this pathway holds certain significance in maintaining their growth motivation. Cultivating future time perspective requires minimal investment, yet it provides resource-constrained female college students in Western China with a broadened future horizon, which has an amplifying effect on their personal growth. Therefore, the statistical significance of this pathway demonstrates that activating future time perspective plays a positive role in overcoming environmental constraints among female college students in Western China.

### 4.3 Examination of the chain mediation effect of psychological capital and future time perspective

The results show that, psychological capital significantly and positively predicts future time perspective. Furthermore, the test of chain mediation effects revealed a significant chain mediation effect of psychological capital and future time perspective on the relationship between basic psychological needs satisfaction and personal growth initiative. According to Self-Determination Theory, when female college students in Western China break through environmental constraints to fulfill their basic psychological needs, this effectively activates their intrinsic motivation and promotes the accumulation of psychological capital. Psychological capital helps them sustain the willingness and behaviors for growth amidst current adversities, while partially transforming intrinsic motivation into future-oriented thinking, thereby enhancing future time perspective. The multiple pathway analysis revealed that psychological capital served as the dominant pathway driving personal growth initiative, whereas the pathway involving future time perspective was relatively modest. This indicates that under the dual constraints of limited resources and environmental pressures, the psychological capital of female college students in Western China is primarily allocated to addressing immediate challenges and fostering present growth. Meanwhile, the results also demonstrate that psychological capital can amplify the promotive effect of basic psychological need satisfaction on future time perspective. This implies that as their self-efficacy, resilience, and optimism levels are enhanced through the satisfaction of basic psychological needs, they gradually develop the psychological impetus and foundational confidence to contemplate the future, thereby creating conditions for more long-term personal growth planning.

Furthermore, the study found that after controlling for individual psychological resources such as basic psychological needs satisfaction, psychological capital, and future time perspective, registered residence (1 = urban, 2 = rural) demonstrated a significant positive effect on personal growth initiative. This indicates that female college students from rural exhibit higher levels of personal growth initiative than their urban counterparts. For female college students from rural Western China, access to college represents a scarce and hard-won opportunity, necessitating highly proactive engagement to fully utilize it. In contrast, their urban counterparts may perceive college as an ordinary life stage. Moreover, rural upbringing has instilled in them resilience, perseverance, and the initiative to independently solve problems—qualities that can be directly applied to foster personal growth during their college experience. In relatively traditional rural western regions, where the allocation of resources is often skewed toward male children (Ma and Wang, 2016), female students who succeed in entering college have likely overcome significant obstacles and developed a strong desire to “prove themselves.” This will promote the development of their personal growth initiative. For these students, higher education represents a primary, or even the only pathway to altering their life trajectories, compelling them to proactively engage in self-improvement and vigorously pursue opportunities. Female college students from rural backgrounds clearly perceive the disparities between themselves and their urban counterparts (Li et al., 2021). This awareness motivates some of them to strive to catch up, as they recognize that redoubling their efforts is necessary to bridge these gaps. Such a mindset of catching up inherently demonstrates personal growth initiative.

## 5 Research implications

This study focuses on exploring the growth mechanisms of female college students in Western China within resource-constrained contexts. It reveals the multiple pathways through which basic psychological need satisfaction influences personal growth initiative via psychological capital and future time perspective. The findings provide a theoretical foundation and practical guidance for designing targeted educational support policies and psychological interventions for this demographic. Personal growth is an active process that can be consciously managed and shaped. For female college students, this can begin by addressing their basic psychological needs, focusing on the fulfillment their needs for autonomy, competence, and relatedness. For instance, they can exercise autonomy in major selection, career planning, and participation in student organizations; build confidence and accumulate a sense of accomplishment through achieving academic goals; and establish high-quality relationships with teachers and peers to experience social support. In doing so, they satisfy their basic psychological needs and lay a psychological foundation for growth and development. On this basis, female college students in Western China can further build a robust internal driving system by investing in their psychological capital (such as fostering self-confidence, optimism, hope, and resilience) and by planning for long-term future goals. This helps them achieve self-growth and developmental objectives even in potentially resource-constrained environments. Universities should integrate the fulfillment of “basic psychological needs” into curriculum design and teacher-student interactions. For instance, offering diverse elective courses to enhance autonomy, and promoting collaborative learning with mentorship programs to strengthen relatedness. University counseling centers can develop group counseling programs aimed at enhancing students’ psychological capital and future time perspective. By strengthening career education efforts, these initiatives can help female college students in Western China effectively translate their psychological capital into a clear blueprint for the future, thereby converting intrinsic motivation into long-term goal attainment. Based on the results of this study, educational administrators and faculty should avoid labeling female students from rural Western China as a “disadvantaged group.” Instead, they ought to recognize that these students have developed strong psychological strengths through overcoming adversities. Support systems for this population should shift from a “deficit-compensation” approach toward a “strength-based” model. Simultaneously, it is essential to provide them with corresponding opportunities and platforms, such as forwarding information about high-quality lectures, internships, and international exchanges, as well as offering specialized workshops to transform their growth motivation into demonstrable competencies. When evaluating the educational outcomes of higher education institutions, education authorities may incorporate students’ positive psychological qualities into the comprehensive assessment system, thereby guiding educational resources to tilt toward promoting students’ holistic development. At a broader societal level, focusing on female college students in Western China will help cultivate more high-caliber female talent who choose to remain in the region. This not only relates to the realization of social values such as educational equity and gender equality but also effectively empowers rural revitalization and sustainable development in western regions, carrying profound significance for narrowing regional development disparities.

## 6 Research limitations and future prospects

### 6.1 Research limitations

This study also has several limitations. First, the cross-sectional design makes it difficult to determine the causal relationships among the variables. Future research could employ longitudinal tracking or experimental interventions to further examine the causal relationships. Second, the study focused on female college students in Western China without including comparative samples from Eastern regions. Given the objective existence of regional development disparities, the psychological characteristics of female college students in different regions need further verification. Additionally, the sampling did not include fourth-year undergraduates, and the potential differences in their personal growth initiative characteristics compared to lower-grade students need to be examined. Future research could enhance the generalizability of the findings by broadening the sampling area and grade range. Finally, although procedural design and statistical tests were employed to mitigate common method bias, the exclusive reliance on self-reported data from the students remains a limitation. Future research could strengthen the validation of the findings by incorporating data from multiple informants.

### 6.2 Future prospects

This study explores the psychological mechanisms underlying the relationship between basic psychological need satisfaction and personal growth initiative among female college students in Western China. Future research could further investigate this mechanism by examining which individual traits or environmental factors may moderate or mediate the pathway from “BPNS → PGI.” Additionally, comparative studies incorporating samples from Eastern China or male college students could be conducted to more precisely examine the uniqueness of personal growth initiative across different populations. Future investigations may also employ longitudinal multi-timepoint tracking designs to examine the dynamic interactive processes between basic psychological need satisfaction and personal growth initiative. Meanwhile, qualitative research methods such as in-depth interviews could be employed to explore how basic psychological need satisfaction promotes personal growth initiative, thereby achieving a deeper interpretation of the statistical data. From the perspective of serving educational practice and social development, future research could focus on validating its application value and exploring new interdisciplinary areas. Intervention programs centered on “basic psychological need satisfaction” and “psychological capital” can be designed and implemented, with their efficacy evaluated through controlled experiments. Meanwhile, further research could explore the predictive effects of personal growth initiative on long-term outcomes (such as career competitiveness and academic innovation capability) among female college students in Western China, thereby directly linking psychological constructs with quantifiable economic, educational, and social benefits.

## 7 Conclusion

The findings of this study indicate that among female college students in Western China, there are significant positive correlations between basic psychological needs satisfaction, psychological capital, future time perspective, and personal growth initiative. Basic psychological needs satisfaction not only directly and significantly predicts personal growth initiative, but also indirectly predicts it through the separate mediating effects of psychological capital and future time perspective, as well as through the chain mediation pathway formed by these two factors.

## Data Availability

The original contributions presented in the study are included in the article/supplementary material, further inquiries can be directed to the corresponding author.
